# Impairment of dendritic cell function and induction of CD4^+^CD25^+^Foxp3^+^ T cells by excretory-secretory products: a potential mechanism of immune evasion adopted by *Echinococcus granulosus*

**DOI:** 10.1186/s12865-015-0110-3

**Published:** 2015-08-13

**Authors:** Ying Wang, Hejun Zhou, Yujuan Shen, Yanjuan Wang, Weiping Wu, Haipeng Liu, Zhongying Yuan, Yuxin Xu, Yuan Hu, Jianping Cao

**Affiliations:** National Institute of Parasitic Diseases, Chinese Center for Disease Control and Prevention; Laboratory of Parasite and Vector Biology, MOH, China, WHO Collaborating Center for Malaria, Schistosomiasis and Filariasis, Shanghai, 200025 PR China

**Keywords:** *Echinococcus granulosus*, Mechanism, Excretory-secretory products, Dendritic cell, Dendritic cell maturation, Cytokine

## Abstract

**Background:**

Cystic echinococcosis, caused by infection with *Echinococcus granulosus*, is one of the most widespread zoonotic helminth diseases. Modulation of host responses is an important strategy used by helminth parasites to promote infection. To better understand the mechanisms adopted by *E. granulosus* to escape host immune responses, we investigated the effects of excretory–secretory products (ES) and adult worm antigen (AWA) derived from adult *E. granulosus* on murine bone marrow-derived dendritic cells (BMDC).

**Results:**

Compared with lipopolysaccharide (LPS), AWA, but not ES, induced BMDC maturation or stimulated BMDC cytokine production and co-stimulatory molecule expression (CD40, CD80 and MHC class II). Furthermore, ES-treated BMDCs pulsed with ovalbumin exhibited reduced co-stimulatory molecule expression in comparison with untreated BMDC, even in the presence of the strong Th1 inducer, CpG. Moreover, we detected the effects of ES-treated DC on T cell activation by an in vitro T cell priming assay. We observed that ES-treated BMDC co-cultured with DO11.10 transgenic CD4^+^ T cells induced the generation of CD4^+^CD25^+^Foxp3^+^ T cells. In addition, in contrast to AWA-treated BMDCs, which had markedly induced IFN-γ secretion and reduced of IL-4 levels in co-cultured T cells, ES-treated BMDCs did not modify their capacity to stimulate IFN-γ or IL-4 production by T cells.

**Conclusions:**

We conclude that ES of adult *E. granulosus* inhibited DC function, impaired the development of Th1 cells induced by CpG, and induced CD4^+^CD25^+^Foxp3^+^ regulatory T cells in an IL-10-independent manner.

## Background

Cystic echinococcosis is a zoonosis with a global distribution that is caused by the dog tapeworm, *Echinococcus granulosus* [1], often resulting in chronic infection and the unlimited growth of hydatid cysts in the liver and lung of humans and domestic animals. *E. granulosus* has a complex life cycle that involves two hosts. The definitive hosts are primarily dogs, which harbor adult worms in their small intestines. Humans and herbivores, particularly sheep, are intermediate hosts of this parasite. Intermediate hosts become infected by ingesting the eggs released in the feces of definitive hosts. Dogs, as the definitive hosts, are pivotal in the transmission of cystic echinococcosis.

Parasitic helminths are capable of maintaining infection for long time periods despite the defense mechanisms of the host. Consequently, these organisms have evolved a wide range of highly elaborate survival strategies, including immunomodulation, antioxidant defenses and resistance to host proteolytic enzymes [2, 3]. However, most of the mechanisms that underlie the downregulation of host responses remain largely unclear, especially at the molecular level, although they are likely to be mediated by proteins found in the parasite somatic extract and excretory-secretory (ES) products (ES) [4, 5].

Dendritic cells (DC) are known to be essential immune cells in innate immunity and in the initiation of adaptive immunity [6, 7]. The shaping of adaptive immunity by innate immunity is dependent on the unique cellular functions of DCs and DC-derived effector molecules, such as cytokines and chemokines. At the interface of the innate and adaptive immune systems, DC senses the invading pathogen and initiates Th1 or Th2 immune responses. Accumulating evidence has demonstrated that pathogens have evolved multiple strategies to subvert the function of DCs. Previous studies have established that the larval stages of *Echinococcus* spp. modulate the function of DCs via ES products. The hydatid cyst fluids and antigen B of *E. granulosus* have been reported to modulate DC differentiation and cytokine secretion [8, 9], and the laminated layer induces the unconventional maturation of DCs [10]. In addition, ES products of *E. multilocularis* larvae induce DC apoptosis and the generation of CD4^+^CD25^+^Foxp3^+^T cells [11]. In our previous work, MHC-II, which is expressed on the surface of antigen presenting cells (APCs), was found to be downregulated during the early stage of *E. granulosus* infection, which suggests a role for ES products in APC function [12]. Overall, the targeting and impairment of DC function is an important immune escape strategy employed by larval *Echinococcus* spp.; however, whether adult ES products or adult worm antigen (AWA) can influence the function of DC remains unknown. The adult parasite stage causes no symptoms in dogs, and this coexistence probably results from the immunomodulation achieved by the adult worm. Therefore, the effects of adult *E. granulosus-*derived products on DC function are an interesting area for research.

In this study, we assessed the effects of treatment of either *E. granulosus* ES or AWA on the maturation of DC and CD4+ T cell activation in response to treated DCs. The aim of this study was to assess the immunomodulatory function of adult somatic cell extracts, which may play an important role in parasite–host interactions. The *in vitro* exposure of bone marrow-derived DC (BMDC) to AWA, but not ES, induced DC maturation and treatment with ES impaired the ability of DC to induce immune responses, even in the presence CpG, a strong Th1 inducer. To the best of our knowledge, this is the first report to shed light on the immunosuppressive effects of adult ES on DC maturation and subsequent T cell activation.

## Methods

### Ethics statement

This study was carried out in strict accordance with the recommendations in the Guide for the Care and Use of Laboratory Animals of the National Institute of Parasitic Diseases, Chinese Center for Disease Control and Prevention. The protocol was approved by the Laboratory Animal Welfare & Ethics Committee (LAWEC), National Institute of Parasitic Diseases, Chinese Center for Diseases Control and Prevention (Permit Number: IPD 2011-006). All surgery was performed under sodium pentobarbital anesthesia, and all efforts were made to minimize suffering.

### Parasites and animals

Adult *E. granulosus* worms collected from dogs were kindly provided by the Qinghai Institute for Endemic Disease Prevention and Control. All experimental mice were maintained under pathogen-free conditions. BALB/c mice were obtained from the Shanghai Laboratory Animal Center, Chinese Academy Sciences and DO11.10 αβ T cell receptor transgenic mice (on a BALB/c background) were purchased from the Model Animal Research Center of Nanjing University.

### Excretory–secretory product and adult worm antigen preparation

Adult *E. granulosus* worms were collected from the intestine by dissection microscopy, washed extensively with sterile PBS (Gibco, California, USA) containing 100 U/ml penicillin G, 100 μg/ml streptomycin (Gibco, California, USA) and cultured at a density of approximately 500 worms per ml serum-free RPMI 1640 medium (Gibco, California, USA) supplemented with 2 % glucose (Sigma-Aldrich, Missouri, USA) and antibiotics for 24 h at 37 °C.

To generate ES, the supernatant was harvested, centrifuged to remove eggs and worm debris and concentrated using a micro-concentrator with a 5 kDa cut-off (Millipore, Nassachusetts, USA). To prepare total adult worm antigen, the remaining adult worms from the culture were washed with PBS and homogenized by sonication. The homogenate was centrifuged (10000 *g*, 5 min) and the soluble fraction collected as AWA. ES and AWA were stored at −80 °C until used. A 2D Quant Kit (Amersham Biosciences, Buckinghamshire, UK) was used to determine the protein concentration in these preparations. The test for endotoxins was performed in AWA and ES, respectively. DCs showed the negligible expression of co-stimulatory molecules after stimulation with the same level of LPS, which suggested that the presence of LPS in purified antigens was lower than its effective concentration.

### Generation of BMDC

BMDC were obtained according to a method adapted from Inaba et al. [13] and Lutz et al.[14]. Briefly, total bone marrow cells were collected from the femur of BALB/c mice and red blood cells were lysed using RBC Lysis Buffer (Beyotime, Shanghai, China). After washing with RPMI-10 (Hyclone, Utah, USA) three times, the bone marrow cells were resuspended in RPMI-10 containing 10 ng/ml granulocyte-macrophage colony stimulating factor (GM-CSF; Peprotech, London, UK) at 1 × 10^6^ cells/ml and cultured for 7 days at 37 °C in a humidified CO_2_ incubator. Cells were fed on days 3 and 5. On day 7, DC clusters were harvested and subcultured overnight to remove adherent cells. Non-adherent cells were collected on day 8 by gentle pipetting and washed CD11c^+^ cells were then purified using a Magnetic-activated Cell Sorting System (Miltenyi Biotec, Auburn, CA) as immature BMDC for use in experiments.

### *In vitro* BMDC stimulation assays

BMDC were resuspended at 10^6^ cells/ml in complete medium containing 10 ng/ml GM-CSF and stimulated with PBS (Gibco, California, USA), lipopolysaccharide (LPS; 100 ng/ml; from *E. coli* strain 0111:B4; Sigma-Aldrich, Missouri, USA), ES (50 μg/ml) or AWA (50 μg/ml) for 24 h. Cells were harvested for flow cytometric analysis of surface marker expression and supernatants were collected for cytokine quantification using an enzyme-linked immunosorbent assay.

### Flow cytometric analysis

For cell surface staining, 10^6^ cells were washed and treated for 30 min on ice with Fc receptor-blocking reagent (FcγIII/IIR Ab; BD PharMingen, California, USA) in sorting buffer (PBS with 1 % fetal calf serum). Blocked cells were then incubated with PE-labeled anti-CD86 monoclonal antibody (mAb; clone GL-1), APC-labeled anti-CD80 mAb (clone 16-10A1), PeCY5-labeled anti-CD40 mAb (clone 3/23) and FITC-labeled anti-MHC class II mAb (A_β_b, clone AF6-120.1) purchased from BD PharMingen. After washing, cells were resuspended in sorting buffer for flow cytometric analysis using a FACSCalibur (BD Biosciences, New Jersey, USA). At least 10,000 gated events were acquired per sample and data analysis was performed using CellQuest or FlowJo software.

### *In vitro* pulsing of BMDC with OVA

BMDC were resuspended at 10^6^ cells/ml in complete medium and treated with ES (50 μg/ml) for 2 h before pulsing with 5 mg/ml OVA for 16 h at 37 °C in the presence or absence of 1 μM CpG during the last 12 h. After pulsing, cells were harvested for the flow cytometric analysis of surface marker expression.

### *In vitro* T cell priming assay

For determination of the T cell-polarizing function of differentially activated BMDC, CD4^+^ T cells were enriched from spleens of DO11.10 transgenic mice by positive selection using anti-CD4 magnetic Beads (Miltenyi Biotec, Auburn, CA) according to the instructions provided by the manufacturer. These cells express a T cell receptor specific for an ovalbumin (OVA) peptide (323-ISQAVHAAHAEINEAGR-339) presented in the context of I-A^d^ MHC class II. Purified CD4^+^ T cells were washed three times and resuspended at 2 × 10^6^ cells/ml in complete medium. BMDC were resuspended at 4 × 10^5^ cells/ml in complete medium and were treated with ES (50 μg/ml), AWA (50 μg/ml) and LPS (100 ng/ml). Untreated DC were used as a control. BMDC were extensively washed and seeded into 48-well culture plates (250 μl/well) in complete medium containing 50 nM OVA323-339 peptide (AnaSpec, California, USA). CD4^+^ T cells were added (250 μl/well) to obtain a final T cell/BMDC ratio of 5:1. Co-cultures were incubated for 48 h at 37 °C before the medium was removed and fresh complete medium containing 10 ng/ml mouse rIL-2 (R&D Systems, Wisconsin, USA) was added. Following an additional incubation for 72 h at 37 °C, cells were harvested, washed and resuspended at 1 × 10^6^ cells/ml. For cytokine analysis by ELISA, 200 μl of this cell suspension were seeded into 96-well flat-bottomed culture plates coated with 5 μg/ml anti-mouse-CD3 mAb (Perprotech, London, UK) and incubated for 48 h at 37 °C prior to harvesting supernatants. For flow cytometric analysis of T cell phenotype, 500 μl of the cell suspension was seeded into 48-well flat-bottomed plates coated with anti-mouse-CD3, incubated for 12 h at 37 °C prior to addition of monensin (2 μl GolgiStop; BD PharMingen, California, USA) and incubated for a further 5 h. Cells were harvested and stained as described above.

### Cytokine quantification

Levels of IL-4, IL-10 and IFN-γ in culture supernatants were determined using an ELISA kit (Biolegend, California, USA) according to the instructions provided by the manufacturer.

### Statistical analysis

Statistical significance was evaluated by one-way ANOVA. Values of *P* < 0.05 were considered to be statistically significant.

## Results

### ES failed to induce DC maturation and robust cytokine production

**Fig. 1:**
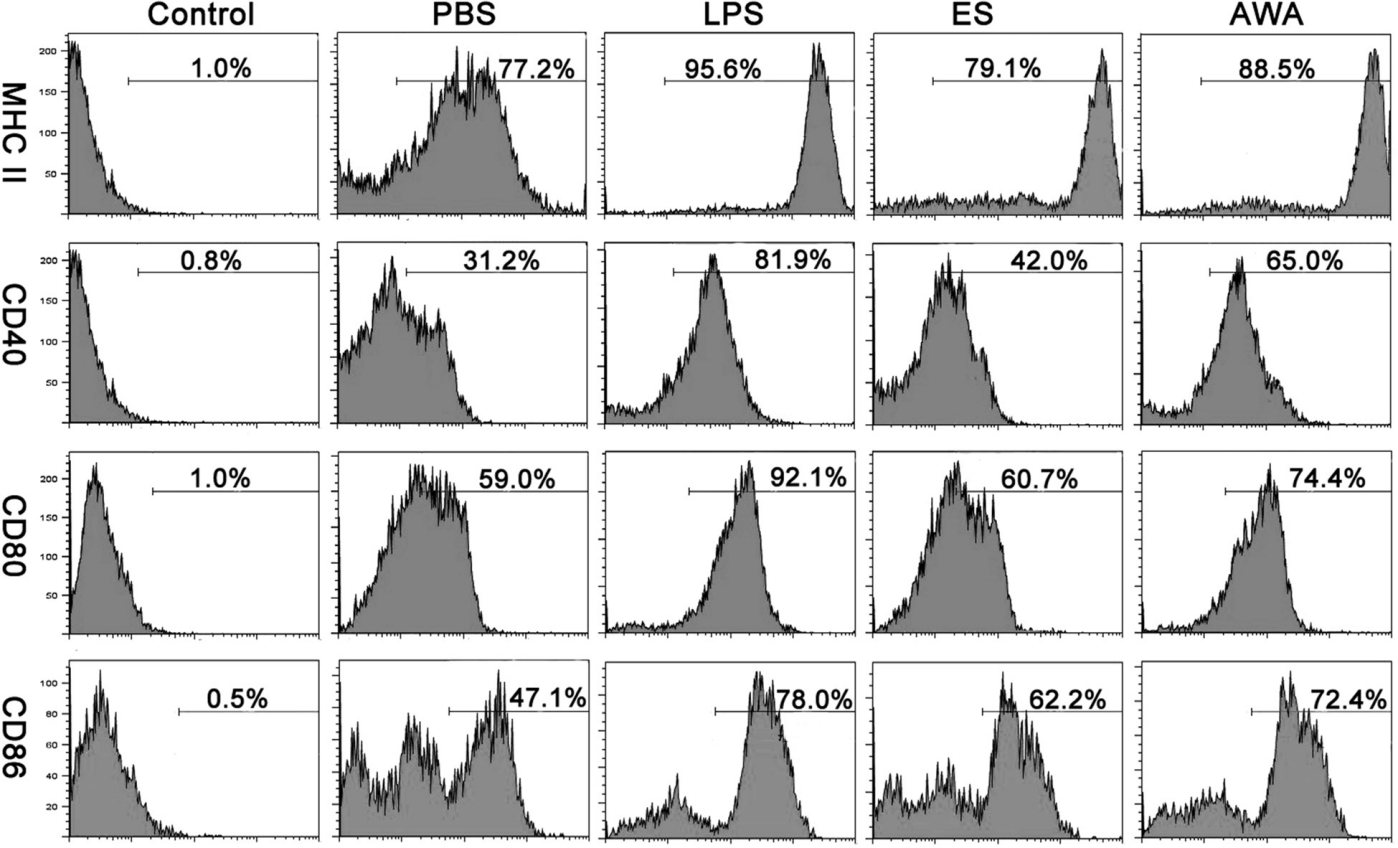
ES from *E. granulosus*, but not AWA, failed to induce DC maturation *in vitro*. Expression of surface markers by BMDC stimulated with PBS, LPS (100 ng/ml), ES (50 μg/ml) or AWA (50 μg/ml) for 24 h. Unstimulated BMDC served as an isotype control of the FC stain. MHC II, CD40, CD80 and CD86 histograms were obtained by gating cells based on positive CD11c staining. The percentage of CD11c^+^ cells expressing the marker is indicated at the top of each histogram

The ability of ES and AWA to induce DC maturation and cytokine production was investigated by using LPS as a positive control and PBS as a negative control. Expression of co-stimulatory molecules (CD40, CD80, CD86 and MHC class II) was analyzed by flow cytometry flowing in vitro stimulation of CD11c^+^ BMDC with ES, AWA, LPS or PBS alone. Interestingly, ES and AWA showed different effects on DC maturation: DC stimulated with AWA expressed higher levels of CD40, CD80, MHC class II and CD86 compared with DC stimulated with PBS (Fig. 1, data are shown in Table 1), manifesting a conventional mature phenotype, similar to that obtained with LPS. In contrast, the expression of co-stimulatory molecules on ES-treated BMDCs was equivalent to that observed in PBS-treated cells.

**Table 1:** The phenotype of DC with different stimuli

N = 3	PBS	LPS	ES	AWA
MHC II	78 ± 1.08	96 ± 6.29	77 ± 2.29	89 ± 2.17*
CD40	32 ± 2.03	82 ± 2.64	41 ± 1.65*	67 ± 1.69*
CD80	59 ± 1.35	93 ± 2.45	61 ± 2.67	75 ± 1.33*
CD86	47 ± 1.58	76 ± 2.27	60 ± 0.95*	72 ± 1.78*

*P < 0.05

**Fig. 2:**
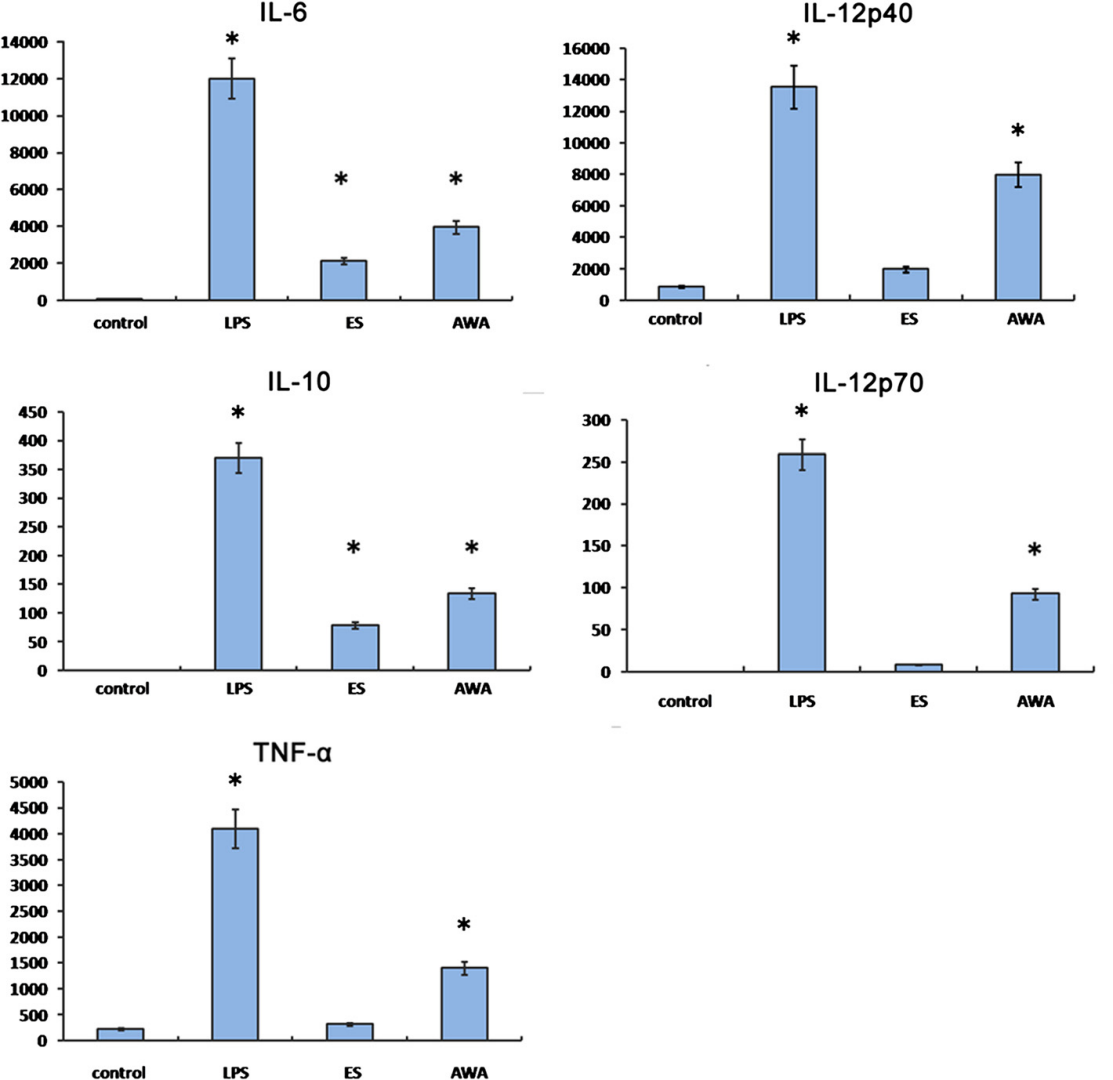
Cytokine production by BMDC stimulated with PBS (as a control), LPS (100 ng/ml), ES (50 μg/ml) or AWA (50 μg/ml) *in vitro*. BMDC were cultured with the indicated stimuli and the production of IL-6, IL-10, TNF-α, IL-12p40 and IL-12p70 was determined by ELISA. Data are expressed as mean pg/ml ± SE from three independent experiments. *P < 0.05 compared to cytokine production in control

Next, we detected the production of cytokines in BMDCs in response to different stimuli. AWA treatment induced the elevation of IL-6, IL-10, TNF-α, IL-12p40 and IL-12p70 levels in BMDCs (Fig. 2) compared to PBS (negative control), but these were lower than LPS-induced cytokine production levels. In contrast, ES treatment only induced marginal IL-6 and IL-10 production compared to negative controls. Thus, AWA rather than ES induced DC maturation and robust cytokine production.

### ES impair TLR ligand-induced DC maturation *in vitro*

**Fig. 3:**
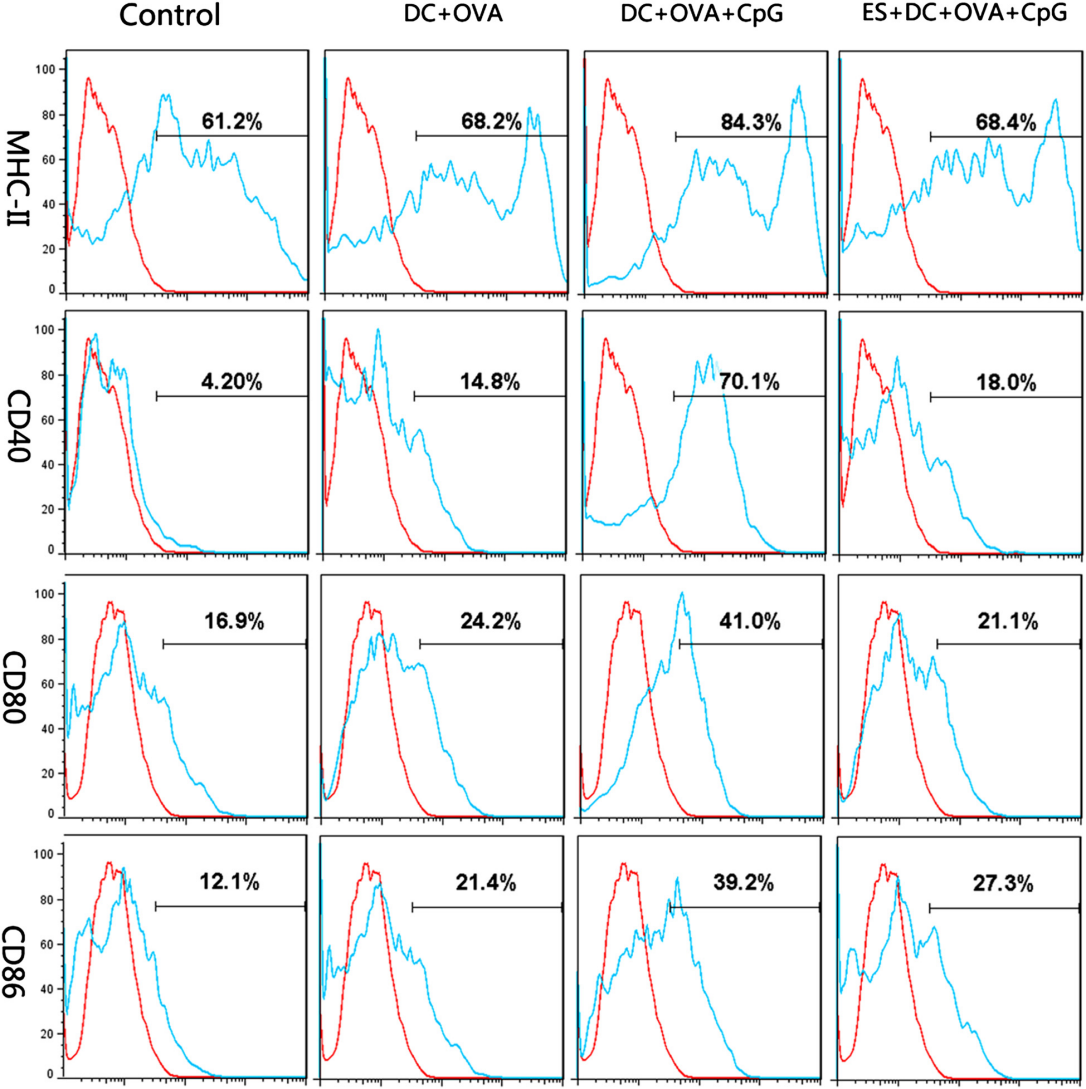
Phenotypic characterization of OVA-pulsed BMDCs. Co-stimulatory molecule expression by control DCs or ES-treated DCs following OVA pulsing. Histograms were obtained by gating cells based on positive CD11c staining. The percentage of CD11c^+^ cells that expressed the markers is indicated at the top of each histogram (the red line and the sky blue line refer to the isotype control and the positive stain, respectively)

To evaluate the effects of ES on DC maturation induced by the Toll-like receptor (TLR) ligand, BMDCs were stimulated with CpG in the presence of ES. As shown in Fig. 3 (data are shown in Table 2), BMDCs pulsed with OVA alone expressed higher levels of CD40 compared with the unpulsed control cells stimulated with PBS. The addition of CpG as an adjuvant during the final 12 h of the OVA pulsing procedure induced higher levels of CD40, CD80, CD86 and MHC class II expression compared with BMDCs pulsed with OVA alone. The ES treatment of DC before they were pulsed with OVA and CpG reduced the expression of the co-stimulatory molecule compared with the responses induced by DCs pulsed with OVA and CpG. These results indicated that ES suppressed DC maturation induced by the TLR ligand.

**Table 2:** The phenotype of OVA-pulsed DCs

N = 3	Control	DC + OVA	DC + OVA + CpG	ES-DC + OVA + CpG
MHC II	62 ± 3.57	67 ± 3.25	85 ± 3.56*	67 ± 2.35
CD40	5 ± 2.14	15 ± 3.67*	70 ± 2.30*	18 ± 3.42*
CD80	17 ± 3.21	24 ± 3.25	42 ± 4.53*	21 ± 3.13
CD86	12 ± 2.75	22 ± 5.73*	40 ± 4.18*	26 ± 2.37*

*P < 0.05

### ES induced CD4^+^CD25^+^ Foxp3^+^ T cell generation

**Fig. 4:**
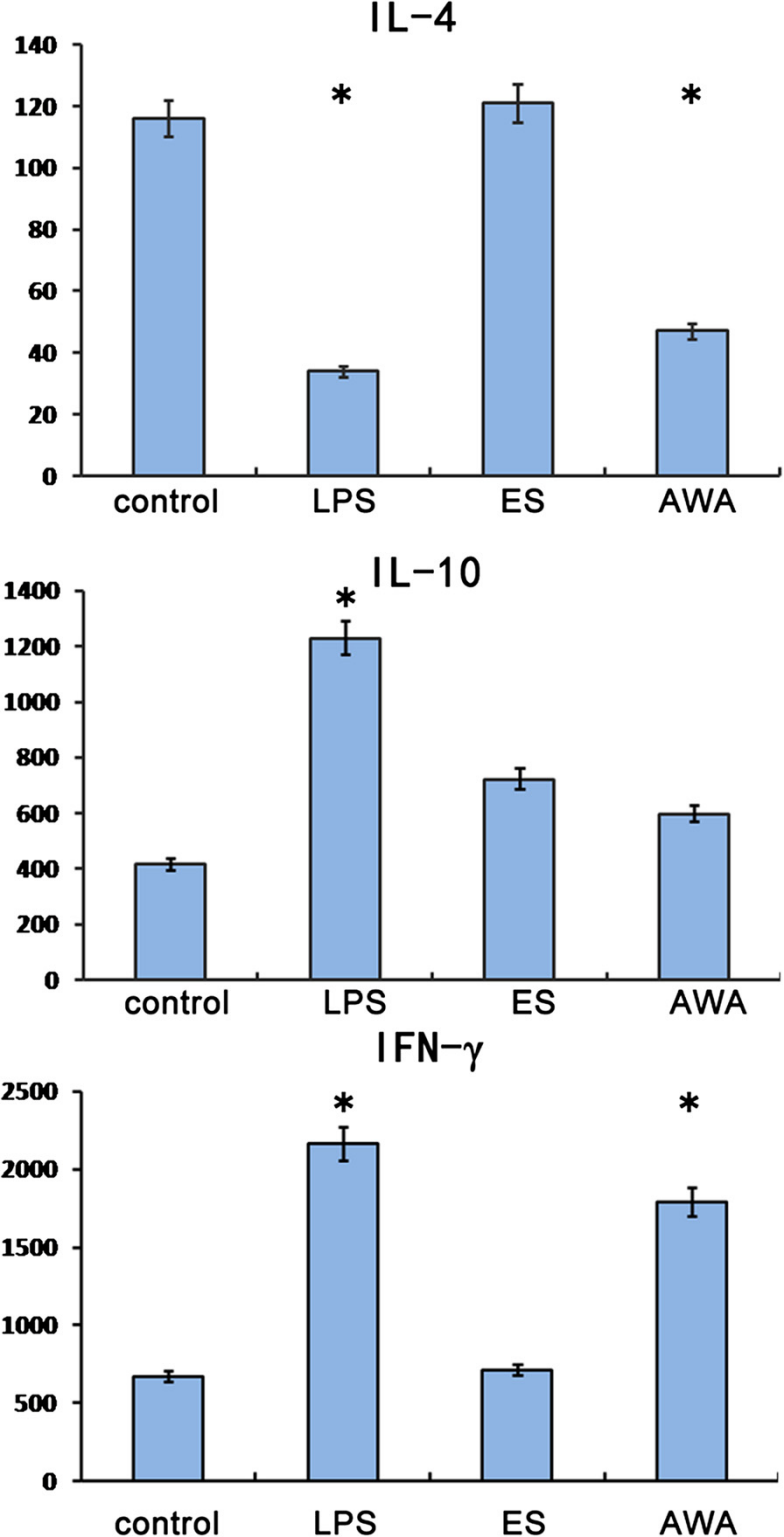
Cytokine production by DO11.10 CD4^+^ T cells cultured alone or co-cultured with LPS-, ES- or AWA-pretreated BMDC *in vitro*. CD4^+^ T cells were purified from the spleens of naïve DO11.10 mice and co-cultured with LPS-, ES- or AWA-pretreated BMDC or untreated BMDC (control) in the presence of 50 nM OVA peptide for 48 h. The final T cell/BMDC ratio was 5:1. After incubation, T cells were reactivated with plate-immobilized anti-mouse CD3 for 48 h. Supernatants were collected for analysis of cytokine production by ELISA. ES–pretreated BMDC failed to enhance cytokine production by CD4^+^ T cells in vitro, while AWA–pretreated BMDC upregulated secretion of IFN-γ and downregulated secretion of IL-4. Spleens from three individual mice were pooled in these experiments. Data are expressed as mean pg/ml ± SE from three independent experiments. *P < 0.05 compared to cytokine production in control BMDC-T cell co-cultures

The effects of ES and AWA on the ability of BMDC to stimulate CD4^+^ T cell cytokine production was also examined using naïve CD4^+^ T cells purified from the spleens of DO11.10 OVA-specific T cell receptor transgenic mice. Preliminary studies demonstrated that stimulation of DO11.10 CD4^+^ T cells co-cultured with control BMDC and 50 nM OVA peptide induced high levels of expression of both IFN-γ and IL-4, and a modest level of IL-10. DCs alone or T cells co-cultured with BMDCs in the absence of OVA peptide produced negligible amounts of these cytokines (data not shown). As shown in Fig. 4, co-culture with LPS-pretreated BMDCs led to a significant increase in the production of IFN-γ and IL-10, and a reduction of IL-4 production in CD4^+^ T cells. Similarly, AWA-pretreated BMDC stimulated CD4^+^ T cells to secrete higher levels of IFN-γ compared with the control. In contrast, the ES pretreatment of BMDCs with ES did not modify their capacity to stimulate IFN-γ or IL-4 production by T cells.

**Fig. 5:**
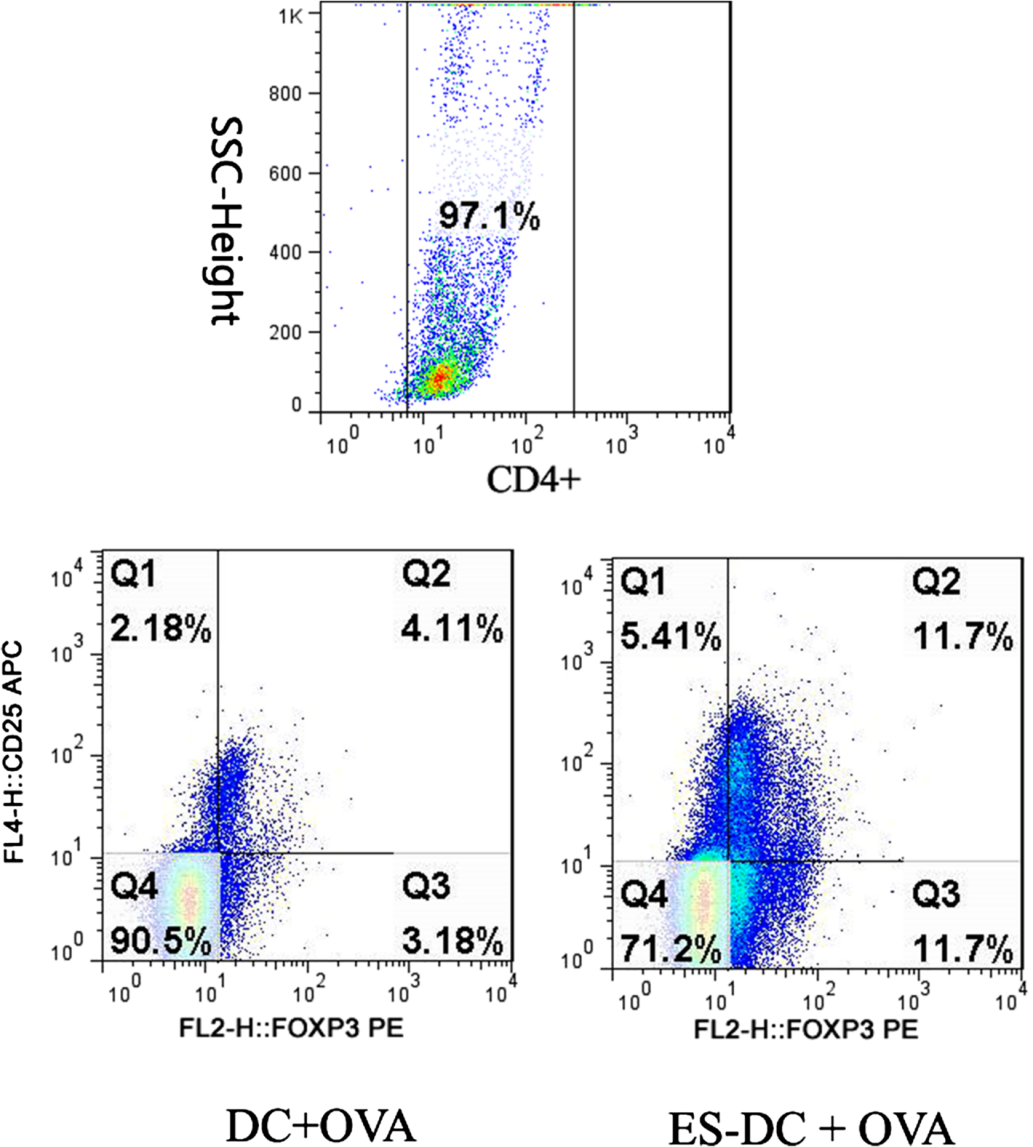
ES induced CD4^+^CD25^+^ Foxp3^+^ T cell generation. BMDC–T cell co-culture was performed as described above in the presence of 50 nM OVA. After initial incubation, T cells were reactivated with plate-immobilized anti-mouse CD3 for 12 h, harvested and analyzed by combined surface and intracellular FACS staining. T cells were gated on the basis of CD4^+^ staining and analyzed for surface CD25 staining and intracellular Foxp3 staining. The percentage of CD4^+^CD25^+^Foxp3^+^ T cells is indicated in the upper right quadrant

Surface expression of CD4 and CD25 and intracellular expression of Foxp3 were also analyzed. ES-pretreated BMDC induced an increase in the percentage of CD4^+^ T cells expressing CD25 and Foxp3 (Fig. 5, data are shown in Table 3). These data suggest that BMDC exposed to ES induced a regulatory population of CD4^+^ T cells that express these two markers.

**Table 3:** The percentage of CD25^+^Foxp3^+^ cells in DC-T cell co-cultures

N = 3	DC + OVA	ES-DC + OVA
CD25^+^Foxp3^+^	4 ± 1.76	11 ± 2.17*

*P < 0.05

## Discussion

In this study, the inability of adult *E. granulosus* ES to induce DC maturation and cytokine production was demonstrated *in vitro* for the first time. ES was shown to suppress DC maturation. AWA was shown to induce a conventional maturation of DC, contrary to the effects of ES. A previous proteomic analysis of the pre-adult stage of *E. granulosus* demonstrated that there are different components and abundance of protein(s)/antigen(s) of parasite during different stages, different hosts, and even during the same stage [15–18]. Although ES and AWA are derived from the same stage of *E. granulosus*, the constituents and abundance of specific protein(s)/antigen(s) are most likely to be distinct. It has been proposed that different types of antigen(s) in ES and AWA may be responsible for differences between the effects of ES and AWA, and especially those antigens that are more abundant. In our preliminary proteomic analysis of the ES and AWA, we observed that the abundance of some proteins are significantly more in the ES, which supported this hypothesis. Some immunomodulatory components and other molecules of *Echinococcus* have been identified, including AgB, elongation factor EgEF-1β/δ, EA21, EgHSP70, EgTeg of *E. granulosus,* and Em2 and Em492 of *Echinococcus multilocularis* [19–28]. In our preliminary research, we identified novel proteins within the ES of adult *E. granulosus* using a two-dimensional electrophoresis, tandem matrix-assisted laser desorption/ionization time-of-flight (MALDI-TOF/TOF) method (data not published). However, the exact nature of the immunomodulatory component(s) in adult *E. granulosus*-derived ES and the associated receptor(s) remain to be elucidated.

Many helminth-derived proteins, glycoconjugates and small lipid moieties have been identified that are thought to contribute to the immunosuppression associated with parasites [29–31]. Our finding of an immature phenotype and weak cytokine production in DCs stimulated with ES is consistent with previous reports of the inability of other helminth products, such as the filarial glycoprotein ES-62 and schistosome-derived soluble egg antigen, to induce DC maturation [32, 33]. In contrast, DC exposed to *Nippostrongylus brasiliensis* ES display increased CD40 and CD86 expression and production of IL-6 and IL-12p40 [29]. These data indicate that some helminth products induce an immature DC phenotype while others induce selective maturation of DC associated with Th2 polarization.

DC recognition of TLR ligands is the first line of defense against pathogens and contributes to activation of adaptive immune responses. The CpG motif induces a highly polarized Th1 response and is generally recognized by TLR9, which is expressed by DC. Therefore, we investigated the capacity of ES to modulate CpG-induced DC activation. In accordance with previous results, CpG induced the expression of CD40, CD80, CD86 and MHC class II, whereas ES suppressed CpG-mediated induction of all these markers, thus demonstrating a strong inhibitory effect on CpG-induced DC maturation. Together, our data suggest that ES have a broadly suppressive effect on DC activation and on the cytokine network that regulates the magnitude of Th1 responses. Thus, we speculate that inhibition of DC maturation and function represents a potential immunosuppressive mechanism used by *E. granulosus*, in a similar way to *Heligmosomoides polygyrus*, *Schistosoma japonicum* and other pathogens [34–36], although further experiments are required to confirm this hypothesis.

Regulatory T cells (Tregs) play an important role in immunosuppression and have the capacity to inhibit innate and adaptive immunity, including both Th1 and Th2 responses [37–39]. We observed in this study that ES-treated DC co-cultured with DO11.10 transgenic CD4^+^ T cells induced the generation of CD4^+^CD25^+^Foxp3^+^ T cells with no increase in IL-4, IL-10 and IFN-γ secretion compared with controls. The phenotype of the CD4^+^ T cell population described here differs from that identified by Vieira et al. [40] and Segura et al. [41], consisting of CD4^+^CD25^+^ T cells that secrete IL-10 and do not express Foxp3, but have regulatory function comparable with that of naturally occurring Tregs. Nevertheless, Vieira et al. demonstrated that, despite lack of Foxp3 expression, IL-10-secreting Tregs were found to produce little IL-2, express high levels of CD25 and inhibit effector T cell proliferation in vitro in an IL-10-independent manner [40, 42, 43]. Segura et al. described a similar effect mediated by an IL-10-dependent mechanism [41]. The lack of IL-10 production in the cells identified in this study suggests that the inhibitory effect of CD4^+^CD25^+^Foxp3^+^ Tregs occurs via an IL-10-independent mechanism.

## Conclusions

In this study, our data provide evidence that ES secreted by adult *E. granulosus* exerts a potent inhibitory effect on the maturation and function of DC, which in turn may drive the differentiation of CD4^+^ T cells into Tregs and suppress cytokine production in a generalized manner irrespective of their Th1 or Th2 phenotypic affiliation. In addition to increasing knowledge of *E. granulosus* infection, our study provide further understanding of the cellular and molecular mechanisms involved in the modulation of host immune responses by helminths and pave the way for the development of anti-helminthic treatment and disease control strategies.

## Abbreviations

APC: Antigen-presenting cells
AWA: Adult worm antigen
BMDC: Bone marrow-derived dendritic cells
CpG: Cytosine guanine dinucleotide
DC: Dendritic cells
ES: Excretory–secretory products
LPS: Lipopolysaccharide
OVA: Ovalbumin
Treg: Regulatory T cells
